# Research on the Cross-Chain Model of Rice Supply Chain Supervision Based on Parallel Blockchain and Smart Contracts

**DOI:** 10.3390/foods11091269

**Published:** 2022-04-27

**Authors:** Xiangzhen Peng, Xin Zhang, Xiaoyi Wang, Haisheng Li, Jiping Xu, Zhiyao Zhao, Yanhong Wang

**Affiliations:** 1 Beijing Key Laboratory of Big Data Technology for Food Safety, Beijing Technology and Business University, Beijing 100048, China; 2030602060@st.btbu.edu.cn (X.P.); wangxy@btbu.edu.cn (X.W.); lihsh@btbu.edu.cn (H.L.); xujiping@btbu.edu.cn (J.X.); zhaozy@btbu.edu.cn (Z.Z.); 2 Key Laboratory of Industrial Internet and Big Data, China National Light Industry, Beijing Technology and Business University, Beijing 100048, China; 3 Beijing Institute of Fashion Technology, Beijing 100048, China; 4 China Academy of Information and Communications Technology, Beijing 100048, China; wangyanhong1@caict.ac.cn

**Keywords:** rice supply chain, cross-chain supervision, smart contracts, blockchain, food safety

## Abstract

Rice is one of the three major staple foods in the world, and the quality and safety of rice are related to the development of human beings. The new crown epidemic, pesticide residues, insect pests, and heavy metal pollution have a certain security impact on the food supply chain. The rice supply chain is characterized by a long life cycle; complex roles in the main links; many types of hazards; and multidimensional, multisource, and heterogeneous information. To strengthen the rice supply chain’s supervision ability under the epidemic situation, a supervision cross-chain model suitable for the complicated data of the rice supply chain based on parallel blockchain theory and smart contract technology was built. Firstly, the data collected in the rice supply chain and different types of data stored in different parallel blockchains were analyzed. Secondly, based on data analysis, a collection/supervision cross-chain mechanism based on “hash lock + smart contract + relay chain”, a concurrency mechanism based on the K-means algorithm and a Bloom filter, and a consensus mechanism suitable for multichain consensus named the Supervision Practical Byzantine Fault Tolerance (SPBFT) were proposed. Furthermore, a cross-chain model of rice supply chain supervision was constructed. Finally, theoretical verification and simulation experiments were used to analyze the operation process, safety, cross-chain efficiency, and scalability of the model. The results showed that the application of parallel blockchains and smart contracts to supervision of rice supply chain information improved the convenience and security of information interaction between various links in the rice supply chain, the storage cost of supply chain data and the high latency of interaction was reduced, and the refined management of the rice supply chain data and personnel was realized. This research applied new information technology to the coordination and resource sharing of the food supply chain, and provides ideas for the digital transformation of the food industry.

## 1. Introduction

Since 2020, COVID-19 has continued to spread [1]. In addition, global food crops have been affected by extreme weather such as droughts and floods, and pests such as locusts and Spodoptera frugiperda [2,3]. The quality and safety of food crops have been seriously threatened. The three major United Nations food and agriculture agencies—the FAO (Food and Agriculture Organization), WFP (World Food Program), and IFAD (International Fund for Agricultural Development)—as well as international organizations such as the WTO (World Trade Organization) and the G20 (Group of Twenty) have called for global cooperation in regional governance to reduce the impact of the epidemic on food security. Rice is one of the world’s three major foods [4]. The global rice output in 2021/22 is expected to exceed 9.5 billion tons. Under the influence of COVID-19, ensuring the quality and safety of rice is an important measure to protect human health. Therefore, since the outbreak of COVID-19, the rice supply chain has been managed more accurately by the relevant rice regulatory authorities and enterprises. Products that do not meet the food safety standards are penalized more severely. To strengthen the supervision of rice quality and safety [5], it ensures the quality and safety of rice under COVID-19.

Agricultural technological innovation is an effective means to strengthen the supervision of the agricultural and food supply chain. The supervision level of the agricultural and food supply chain is improved by using new technologies. It can also promote the digital transformation of agricultural products and food, and the agricultural products and food quality and safety can be realized [6,7]. The parallel blockchain is an organic combination of parallel intelligent theoretical methods and blockchain technology. It is committed to adding computational experiments to the current blockchain technology through the parallel interaction and coevolution of the actual/artificial blockchain system [8]. It realizes the management of and decision making by the blockchain system by combining description, prediction, and guidance [9]. Agricultural and food supply chain data is complex, and frequently interacts. By referring to the parallel blockchain idea, the storage pressure of the blockchain is reduced, the transaction volume of the blockchain system is increased, and the storage cost of agricultural and food supply chain data is reduced. The application of a parallel blockchain can help the efficient management of complex information in the whole life cycle of the agricultural and food supply chain. A smart contract is a computer program with a status and a conditional response running on a distributed ledger. The contract completes the encapsulation, verification, and execution of the complex behaviors of distributed nodes through preset rules, and it achieves the functions of information exchange, value transfer, and asset management [10,11,12]. In recent years, the combination of blockchain technology with artificial intelligence, big data, 5G, and the industrial internet have been explored by researchers to strengthen regulatory capabilities, which has been mainly reflected in the following aspects [13,14,15]. Firstly, artificial intelligence (AI) and smart contracts were combined to solve the problem of redundancy of blockchain information and improved supervision efficiency [16,17]. Secondly, blockchain technology and big data technology were combined to unify different data sources and realize unified data supervision [18,19]. Thirdly, blockchain technology and 5G technology were combined to solve the problem of slow real-time data transmission [20,21]. Fourthly, the blockchain was combined with the industrial internet, and the precise traceability of regulatory information was achieved through identification analysis [22]. Compared with the traditional agricultural and food supply chain supervision model, the “blockchain+” model can ensure the safety and credibility of the data in the agricultural and food supply chain. The credible traceability and precise accountability of the agricultural products and food data can be realized, thereby improving the supervision of the agricultural and food supply chain efficiency and authenticity.

The rice supply chain is characterized by complex links, diverse data types, and long life cycles. The application of the blockchain and smart contracts has promoted the digitization and intelligence of the rice supply chain, and the supervision of the rice supply chain by the regulatory authorities has been improved to a certain extent. However, as the amount of data has increased, the application of a blockchain and smart contracts in the supervision of the rice supply chain has encountered the following shortcomings.

- The research on blockchains in the rice supply chain is mostly on single-link blockchains such as the “production blockchain”, “processing blockchain”, and “storage blockchain” [23,24,25]. Blockchain research in the rice supply chain is mostly the in the mode of “blockchain + local database” or “blockchain + cloud database” [26]. It is difficult for data to be interconnected in a timely and effective manner between all links of the rice supply chain. There are security risks in the data interaction between the blockchain and the local database.
- Due to the numerous links in the rice supply chain and the huge actual circulation of rice, the amount of data generated by the rice supply chain is huge [27,28]. The blockchain itself has limited storage space, and the single-chain architecture cannot afford the huge amount of data in the rice supply chain [29]. The single-chain architecture has problems such as high latency and high storage costs [30].
- The data storage for the rice supply chain is decentralized, and the basic information, harmful substance information, and personnel identity information of each link are weakly correlated [31,32,33,34]. The management of rice supply chain data is sloppy, and regulators can only supervise rice data, so it is difficult to effectively supervise related fraudulent activities of enterprises [35,36,37].

To solve the weak availability of the blockchain and smart contracts in the rice supply chain in view of the unique architecture and data flow characteristics of the rice supply chain, this paper builds a cross-chain supervision model of the rice supply chain based on parallel blockchains and smart contracts. The main contributions of this paper are listed below.

- It is difficult to effectively interconnect each link of the rice supply chain, and the data interaction between the blockchain and the off-chain database is characterized by security risks [38,39]. We designed a multichain model of “main chain + parallel chain “suitable for rice supply chain supervision based on parallel blockchains and smart contracts. This model allowed the whole life cycle data of the rice supply chain to be stored on the blockchain, and the convenience and security of data interaction between various links were improved.
- In view of the many links in the rice supply chain, the participants are complex, and the rice circulation is huge [40,41,42]. A cross-chain mechanism based on a hash locking mechanism, smart contract technology, and relay chain architecture was designed. A concurrency mechanism based on the K-means algorithm and a Bloom filter was designed. An SPBFT consensus mechanism based on the PBFT consensus mechanism was designed. These mechanisms effectively reduced the delay in complex data interaction in the rice supply chain, and greatly reduced the cost of data storage.
- In view of the characteristics of scattered data storage in the rice supply chain, and since it is difficult for regulators to supervise corporate behavior, four types of smart contracts were customized. The design of these smart contracts strengthened the coupling between rice supply chain data, basic information, harmful substance information, and personnel identity information, and a refined management of rice supply chain data and personnel was realized.

Faced with the unique link structure and complex flow data of the rice supply chain, the existing blockchain research has strengthened the management capability of rice supply chain information to a certain extent. However, the application of and research on the existing blockchain and smart contracts in the rice supply chain are still immature, with low security of data interaction, poor performance of comprehensive management of the whole supply chain, and weak performance of all types of data management, making it difficult to manage the rice supply chain efficiently and precisely. Based on parallel blockchain theory and smart contract technology, this paper designed and used a new “parallel chain + main chain” architecture in the rice supply chain. The cross-chain mechanism, concurrency mechanism, and SPBFT consensus mechanism were also designed to serve in the operation of the rice supply chain supervision model. This paper solved some limitations of the blockchain in rice supply chain information management, and realized the precise management of rice supply chain information.

The rest of the paper is organized as follows. Section 2 is a literature review. Section 3 analyzes the supervision information of the rice supply chain and divides the parallel chains. Section 4 designs the cross-chain mode of rice supply chain supervision, including the design of the cross-chain framework of rice supply chain supervision and the cross-chain mechanism, concurrency mechanism, and consensus mechanism. Section 5 shows the results, and through analysis of the model operation process, safety, efficiency, and extensibility, shows the supervision effect of the model on the rice supply chain. Section 6 contains the conclusion and suggestions for future work.

## 2. Literature Review

The cross-chain regulatory model of the rice supply chain based on parallel blockchains and smart contracts requires higher requirements for latency, privacy, security, convenience, and granularity of data management for cross-chain data interaction in the rice supply chain. In this section, we have compiled some of the latest research on the use of the blockchain for agricultural products, as well as for food in recent years, as shown in Table 1.

In a theoretical study of blockchain-based frameworks or models for agricultural products and food management, [19] noted that the use of the blockchain in food and beverage supply chains may offer benefits by improving food safety, supplier reputation, visibility of small farmers, efficiency in tracing food contamination sources, transparency, and accountability. Ref. [20] explored the challenges faced by typical management systems, such as food safety, food fraud, and inefficient processes, as well as ethical aspects such as fair trade, animal welfare, and the environmental impact of food production. The authors pointed out that the use of blockchain-based systems to manage supply chains offers significant benefits, such as faster and more reliable traceability. Ref. [25] pointed out that digital transformation of agricultural and food supply chains promises a traceable, transparent, trustworthy, and intelligent ecosystem through blockchain-based smart contract technology. Ref. [29] pointed out that the blockchain has great potential to improve the performance of food supply chain traceability by providing security and full transparency. The aforementioned studies explored the advantages of blockchain applications in agricultural and food management frameworks or models, but the ability of the blockchain to handle the huge volume of data in agricultural and food supply chains has not been explored.

In research on the blockchain and smart-contract-based agricultural products and food information management, [1] proposed a blockchain and an approach based on a deep-learning model for food market regulation: stacked autoencoders. The authors provided a basis for market regulation by predicting consumers’ emotional tendencies. Ref. [24] proposed a blockchain-based solution to manage a country’s strategic grain reserves using Hyperledger Fabric software. Ref. [32] combined a cryptographic algorithm, timestamp technology, consensus algorithm, and sidechain technology of the blockchain with the rice supply chain. A federated chain was built between upstream and downstream companies in the supply chain, including producers, suppliers, and vendors, to create secure and efficient supply chain information management. Ref. [35] pointed out that the use of the blockchain in the food supply chain was beneficial to reducing management costs, as well as improving management efficiency. Ref. [38] proposed a hierarchical multidomain blockchain network structure and secondary verification mechanism for food supply chain supervision, combining the characteristics of the blockchain such as distribution, transparency, collegiality, and the practical need for regional autonomy. The above research focused on changing the traditional centralized management model through the decentralized feature of the blockchain, which was used to enhance the information-control capability of agricultural products and food. However, the granularity of information management for agricultural products and the food supply chain has high requirements, and the ability of the blockchain to manage complex data interaction requests is still a problem to be solved.

In research on the blockchain and smart-contract-based agricultural products and food information traceability, [21] proposed a blockchain-based traceability system for agricultural products to monitor data on the blockchain, as well as to improve security and traceability of agri-food businesses. Ref. [22] proposed a complete blockchain-based agricultural and food supply chain solution to ensure traceability, trust, and delivery mechanisms in the agricultural and food supply chain. Ref. [33] proposed a blockchain-based traceability architecture for minimizing the risk of COVID-19 and bacteria, fungi, and parasites in the frozen meat supply chain. The aforementioned study utilized the characteristics of the blockchain such as nontamperability and transparency to improve the information traceability of agricultural products and foods, but the efficiency of this information traceability still needs to be improved.

In the area of blockchain-based applications for the integration of agricultural products and food with the Internet of Things, Ref. [17] developed a supply chain traceability system framework based on the blockchain and radio frequency identification technology for tracing food products in the supply chain. Ref. [28] proposed a new approach to apply blockchain and IoT technologies to support the traceability of farm produce sources by collecting data through sensors to be stored in the blockchain and by using smart contracts for bookkeeping. Ref. [30] proposed an agricultural supply chain management architecture using the blockchain and IoT to address the storage and scalability optimization, interoperability, security, and privacy issues, as well as personal data privacy and storage issues in current single-chain agricultural supply chain systems. The above study integrated technologies such as IoT with the blockchain for application, which were then used to improve the information traceability and management of agricultural products and food, which provided an application idea for future blockchain applications.

In this paper, we built a cross-chain supervision model for the rice supply chain based on a parallel blockchain and smart contract, and adopted a new architecture of “main chain + parallel chain” to extend the performance of the blockchain. We designed a cross-chain collection mechanism and a cross-chain supervision mechanism for the rice supply chain to ensure the privacy and security of the cross-chain data. A concurrency mechanism based on the K-means algorithm and a Bloom filter was designed to cope with the high frequency of data interaction in the rice supply chain and reduce the latency of the model. An SPBFT consensus mechanism was also designed to serve the consensus requests between the nodes of the new architecture. This study can improve the information supervision capability of the rice supply chain and ensure the food quality and safety of rice.

## 3. Analysis of Supervision Information on the Rice Supply Chain and Division of Parallel Chains

**Figure 1:**
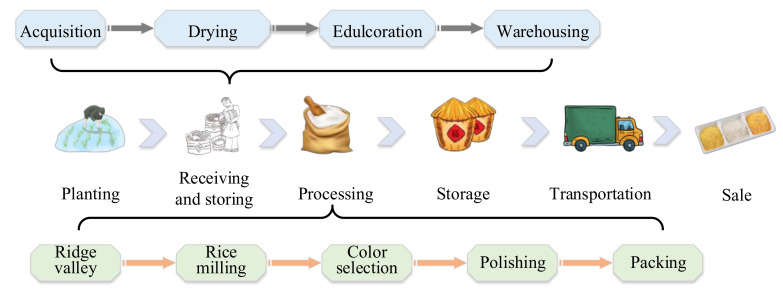
Schematic diagram of the classification of rice supply chain links.

To strengthen the supervision of the rice supply chain and reduce the flow of problematic rice into the market, a cross-chain supervision model of the rice supply chain based on the blockchain and smart contracts was designed. Firstly, we divided the rice supply chain into six typical links: planting, receiving and storing, processing, storage, transportation, and sales. The receiving and storing link included four subnodes: acquisition, drying, edulcoration, and warehousing. The processing node had five subnodes: ridge valley, rice milling, color selection, polishing, and packaging. Figure 1 shows the classification of the rice supply chain links. Secondly, the rice supply chain supervision cross-chain model architecture was divided into three different types: main chain, parallel blockchain, and relay chain. Among them, the main chain nodes were various regulatory agencies, on-chain companies, and consumers; the parallel blockchain was the data storage chain; and the relay chain was the data cross-chain transfer chain. Finally, we sorted the different data generated during the entire life cycle of the rice supply chain and preliminarily divided it into nine parallel chains for storage.

Regarding the different types of data stored in different parallel blockchains, nine parallel blockchains were stipulated that corresponded to hazardous substance information, corporate information, consumer information, regulatory agency information, transaction records, cost information, data interaction records, health records, etc. (Table 2). More than 200 types of key data were compiled based on relevant national/local/industry standards and actual data generated in each link of the rice supply chain. The data covered the entire process for rice, from planting to edible circulation. The data in this paper was collected from IoT devices such as RFID, NFC, mobile phones, computers, and GPS. After the rice data were stored in the blockchain through the collection cross-chain mechanism designed in this paper, the rice supply chain information supervision model established based on parallel blockchain and smart contract technology managed the collected data. The model in turn enabled trusted supervision of the data.

Based on the analysis of key data in the rice supply chain, we organized the data privacy issues arising from the rice supply chain. Data owners fell into three categories: businesses, regulators, and consumers. For enterprises there were six blockchains: parallel blockchains I, II, V, VI, VIII, and IX. Among them, the data contained in the four parallel blockchains II, V, VIII, and IX were shared data, and the related harmful substance information and cost information in the parallel blockchains I and VI were nonpublic data. For regulators, they owned data stored on parallel blockchains IV and VII. The data contained in parallel blockchain IV were shared data, and the data contained in parallel blockchain VII were private data. For consumers, their own data were in parallel blockchain III. The blockchain data is shared data. In terms of access rights, enterprises and consumers can access all shared data after verifying their identities. When the supervisory authority exercises supervisory powers, the corresponding data is accessed according to the authority it has. Based on the key data for the supervision of the entire rice supply chain, the authority of the relevant supervisory authorities of the rice supply chain (Table 3) were classified. Given the different nature of each regulatory department, we regulated the parallel blockchains that each department had the right to supervise and access to ensure the privacy and security of data at the application level.

The links in the rice supply chain are complex, and the amount of rice data is huge. Different data types produced by the rice supply chain into different parallel chains were categorized and stored to improve the efficiency of data supervision by the supervisory authority. Since the chain nodes of various regulatory agencies were distributed in the main chain, a rice supervision framework was designed to solve the problem of cross-chain interaction between the main chain and the parallel blockchain. This framework could realize cross-chain intercommunication between the main chain and the parallel blockchain. Based on this, credible data interaction, credible data collection, and credible data supervision in the rice supply chain were solved by this framework.

## 4. Design of the Cross-Chain Model

### 4.1. Cross-Chain Framework of Rice Supply Chain Supervision

**Figure 2:**
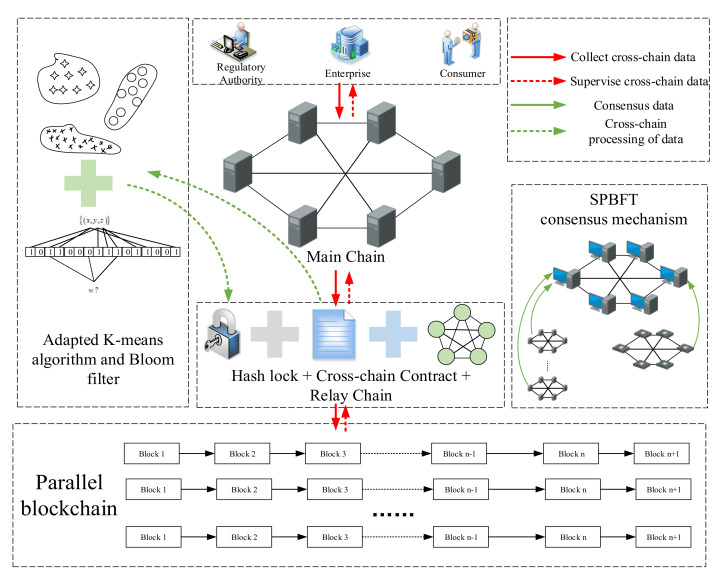
The cross-chain framework of rice supply chain supervision.

The quality and safety of rice are closely related to human health. Strengthening the supervision of the entire rice supply chain is one of the important measures to ensure the quality and safety of rice. At present, most of the research on food quality supervision based on the blockchain is on the single-chain mode, in which a storage mode of “blockchain + cloud database” is adopted to store the supervised data. However, the rice supply chain is characterized by complex links, long life cycles, and a large variety of data. The traditional single-chain model cannot achieve complete decentralization of the data; therefore, it cannot guarantee the absolute security of data storage. In addition, with increases in time and rice business, the data generated by the rice supply chain will steadily increase, which will lead to a continuous increase in the computing resources required for supervision. We proposed a cross-chain framework for rice supply chain supervision to solve the above problems. Figure 2 shows the cross-chain framework of rice supply chain supervision.

In the cross-chain framework of rice supervision we designed, three groups of people are served; namely, regulatory authorities, enterprises, and consumers. An organic combination of hash lock, cross-chain contract, and relay chain was adopted in the cross-chain mechanism. Among these, the cross-chain contracts were divided into CCSC-A (collection cross-chain smart contract A), CCSC-B (collection cross-chain smart contract B), SCSC-A (supervision cross-chain smart contract A), and SCSC-B (supervision cross-chain smart contract B). At the same time, the K-means algorithm and Bloom filter were used to facilitate the data transmission between the main chain and the parallel blockchain, and the SPBFT consensus mechanism was designed to reach a consensus between the main chain and the parallel blockchain. At the bottom of the frame is the parallel blockchain group, which mainly realized the branched storage of data. We divided the data throughout the entire framework into four categories according to types, namely collecting cross-chain data, supervising cross-chain data, consensus data, and cross-chain processing data. A cross-chain mechanism combining a hash lock, smart contract, and relay chain was designed to resist cross-chain attacks in the cross-chain process and ensure the credible transmission of data; a concurrency mechanism based on the K-means algorithm and a Bloom filter was designed to avoid data in the cross-chain process being blocked and to improve the efficiency of the data cross-chain; while the SPBFT consensus mechanism was designed to reach a consensus for each node between the main chain and parallel blockchain.

### 4.2. Mechanism

#### 4.2.1. Cross-Chain Mechanism Based on Hash Lock, Smart Contract, and Relay Chain

(1) Collection cross-chain mechanism

**Figure 3:**
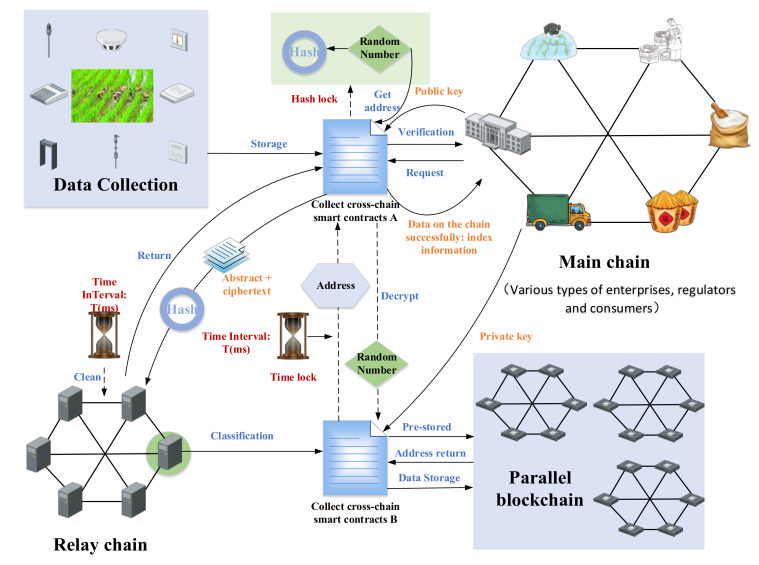
Schematic diagram of collection cross-chain mechanism.

Participating companies, regulatory authorities, and consumers involved in each batch of the rice supply chain are involved in the data interaction of multiple chains when collecting data in real-time. To ensure data security in the process of the data cross-chain, we designed a collection cross-chain mechanism, as shown in Figure 3.

First, various companies, regulatory agencies, and consumers must be certified in the supply chain. When data needs to be stored on the chain, the user sends a request to CCSC-A. After CCSC-A is authenticated, storage permissions will be opened to users. The data collected by the collection equipment is standardized and processed to form *D* (all data) and *Ds* (data digest). CCSC-A encrypts *D* with a hash lock to form a ciphertext *Dn*. Hash lock is described as N (random number) and is randomly generated, and then *H*(*N*) (a unique hash value of *N*) is generated, where *N* is the decryption key of *D*, and *H*(*N*) is the dongle of *D*. CCSC-A obtains *pk_p_* (user’s public key), and it uses *pk_p_* to encrypt *Dn*. *Ds* and *H*(*N*) to form DATA (data packet). CCSC-A transmits the DATA fragments to the relay chain; that is, each node only owns a part of the DATA. Then, *T* (time lock) is designed. Within the range of *T*, each node of the relay chain reaches a consensus and randomly selects a node to restore DATA through the game. In the restoration process, each node endorses the DATA slice. When time *T* ends, each node will automatically encrypt DATA, and the encryption result is returned to CCSC-A for verification. When CCSC-A receives the results returned by more than 51% of the nodes in the relay chain, it can be determined that the DATA slice in the relay chain has been processed. After the endorsement of each node in the relay chain is completed, CCSC-B obtains *sk_p_* (user’s private key) for decryption, and *Ds*, *Dn*, and *H*(*N*) are obtained. CCSC-B calls the concurrency mechanism to process *Ds*, then prestores *Ds* and returns the address *AD*. After that, CCSC-B uses *H*(*N*) to encrypt *AD.* CCSC-A calls CCSC-B, the contract enters *N*, decrypts to obtain *AD*, and stores it on the main chain. After CCSC-B obtains *N*, it decrypts *Dn*, and obtains *D*. The same operation is performed on *D* to obtain the data digest, which matches with *Ds* to verify whether *D* has been tampered with. Finally, the data is stored on the chain according to *AD*. The above data interaction needs to be done within *T* time and only once, otherwise CCSC-A will terminate this data upload. Regarding the security of the relay chain, the endorsement of each relay chain in the cross-chain data can be used to monitor and review problem nodes.

Algorithms 1 and 2 shows the pseudo-code design of the CCSC-A and the CCSC-B mentioned in the cross-chain collection process. The detailed logic design of Algorithms 1 and 2 is provided in Appendix A. Among them, *H* (user) is the user’s only hash, *Y* is the storage success certificate, and *F* is the storage failure notification. There is a mutual calling relationship between CCSC-A and CCSC-B. This mechanism guarantees storage security during the data storage process through relay chains, hash locks, and cross-chain smart contracts. It can respond to attacks promptly and realize the trusted data storage of this model.
**Algorithm 1** Collect cross-chain smart contract A(CCSC-A)Input: *H*(*user*); *D*; *Dn*; DATA; *Ds*; *T*; *AD*; CCSC-B; *pk_p_*; *Y*; *F*; 1:func Certification(*H(user)*) (*D*) // Data acquisition module 2:func Hash lock(*D*)// Data encryption module 3:func Slice(*DATA*)// Data fragmentation module 4:func Game(Relay chain (node))// Game module 5:func Get(*AD*)// Storage module

**Algorithm 2** Collect cross-chain smart contract B(CCSC-B)Input: DATA; CCSC- A; *N*; *skp* func Get-Crack (DATA,*skp*)// Obtain DATA and use user *skp* to decrypt to obtain *Ds*, etc. func Pre-stored(*Ds*)// Pre-storage based on data summary, get *AD* func Get(*N*)// Obtain *N* through data interaction, and generate *Y*/*F* to CCSC-A func Storage(*D*)// Store according to the pre-stored address

(2) Regulatory cross-chain mechanism

**Figure 4:**
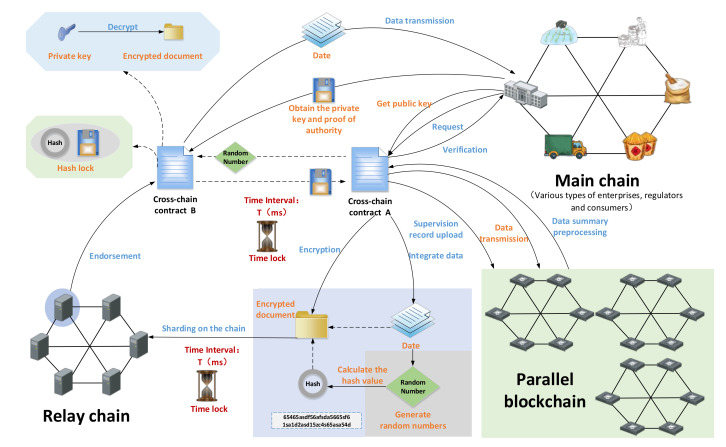
Schematic diagram of supervision cross-chain mechanism.

When the main chain supervisory authority needs to supervise the data, the supervisory data may involve data interaction between multiple chains. To ensure that the data is not tampered with or misappropriated during the transmission process, a supervision cross-chain mechanism was designed to ensure the safety of supervision data transmission. Figure 4 shows this supervision cross-chain mechanism.

The supervisory authority node on the main chain sends a supervisory request to SCSC-A and submits the required supervisory cross-chain data request to SCSC-A. SCSC-A verifies its unique hash value, and if the permissions match, the request is passed. The supervision of cross-chain data requests is standardized by SCSC-A. The required data may exist in multiple subchains. Therefore, SCSC-A uses a concurrent mechanism to preprocess the data to be viewed by the regulatory agency. SCSC-A supervises the location of the cross-chain data stored in parallel blockchains and puts the request into the cross-chain waiting queue of each parallel blockchain. Each parallel blockchain sends data to SCSC-A, and the contract integrates it to form *Data* (supervised cross-chain data). A random number *N* is formed, and a unique hash value *H*(*N*) is generated. *H*(*N*) encrypts *Data* to form *Dn*. At the same time, the time lock *T* is set by SCSC-A. SCSC-A calls the *pk_p_* of the main chain supervision department to encrypt *Dn* and *H*(*N*) to form DATA. Then the fragments are saved into the relay chain. Each node of the relay chain endorses the slice data. Then, the sorting node through the SPBFT consensus mechanism is selected randomly. The DATA is restored, and it is transmitted to SCSC-B. When the time interval *T* arrives, each node of the relay chain randomly encrypts and locks the data in the chain, and returns the encrypted hash value to SCSC-A. When SCSC-A receives feedback from more than 51% of the nodes in the relay chain, it can be determined that the cross-chain data in the relay chain has been destroyed. SCSC-B obtains *sk_p_* and *S* (proof of authority) from the regulatory authority. *Dn* is obtained after SCSC-B uses *sk_p_* to decrypt DATA. SCSC-B uses the same *H*(*N*) to encrypt *S*. After calling SCSC-B, SCSC-A decrypts with *N* to obtain *S* and uploads it to the parallel blockchain to record this data interaction. Finally, SCSC-B obtains the random number *N* at the same time. After the contract decrypts *Dn*, *Data* is obtained, and is sent to the supervisory authority. At the same time, the data slice in the relay chain is automatically destroyed. After SCSC-B decrypts, it sends a decryption success message to SCSC-A, and the data cross-chain is completed this time. If SCSC-A does not receive a message within the time range *T*, the cross-chain fails this time, and SCSC-A initiates emergency measures to encrypt and block all designed cross-chain data.

The supervisory cross-chain mechanism relies on the mutual calls between SCSC-A and SCSC-B to realize the safe transmission of data and ensure the security of the supervisory data. To this end, we designed the smart contract pseudo code, where *H*(*S*) is the hash of the regulatory authority, *V* is the notification of successful decryption, and *IR* is the data interaction record. Algorithm 3 shows the pseudo-code of SCSC-A, and Algorithm 4 shows the pseudo-code of SCSC-B. The detailed logic design of Algorithms 3 and 4 is given in Appendix A.

**Algorithm 3** Supervise cross-chain smart contract A(SCSC-A)Input *H*(*S*); *Ds*; *PA*; SCSC-B; *V*; *IR* 1: func Certification(*H*(*S*))//Verify Permissions 2: func Pretreatment(*D*)//Cross-chain request preprocessing 3: func Integration(*Data*)//Form a supervised cross-chain data *Data* 4: func Slice(*DATA*)//*DATA* slices are stored in the relay chain 5: func Storage(*IR*)//Interactive record storage 6: func Destroy(*S*)//Call SCSC-B, decrypt, and get *S* 7: func Determine(*V*)//Time lock trigger design, including trigger conditions and trigger results

**Algorithm 4** Supervise cross-chain smart contract B(SCSC-B)Input *Data*; *N*; SCSC-A; *skp*; *T*; *DATA* func Decrypt(*DATA*)//Decrypt *DATA* with *skp* func Get(*N*)//Get *N*, pass *S* func Get(*Data*)//Decrypt to get *Data* func Transport(*Data*) func Self-defense(*T*, SCSC-A)//Anti-attack design

#### 4.2.2. Concurrency Mechanism Based on K-Means Algorithm and Bloom Filter

**Figure 5:**
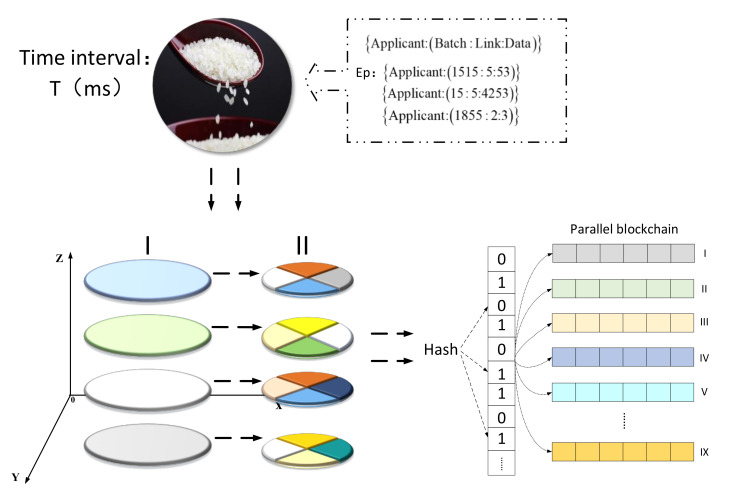
Schematic diagram of concurrency mechanism.

A data cross-chain is prone to high concurrency problems. First of all, when the main chain node needs to cross-chain storage and the number of cross-chain invocations is too large at the same time, the amount of data that the smart contract needs to process far exceeds the designed amount of data, which is likely to cause data cross-chain blockage, which can seriously cause cross-chain “paralysis”. At this time, a “traffic policeman” is needed to command the data and realize the orderly cross-chain of data. To deal with the problem of high concurrency of cross-chain data, the K-means clustering algorithm was used to preprocess the cross-chain data to achieve orderly cross-chain data. In addition, each time the cross-chain data was required to involve multiple parallel chains. As time increased, as well as the expansion and subdivision of cross-chain data types, it would cause the amount of calculations needed to confirm the storage location of cross-chain data to increase linearly. We applied a Bloom filter for processing, thereby greatly reducing the amount of calculations needed. Through the organic combination of the K-means clustering algorithm and the Bloom filter, the problem of data cross-chain concurrency was solved. Figure 5 shows the concurrency mechanism.

First, the required cross-chain data packet Data is defined, with each packet containing multiple cross-chain data, as shown in Equation (1):

(1) $$Data=\left\{\begin{array}{c} \left\{\mathrm{Applicant}(121,5,123)\right\}\\ \left\{\mathrm{Applicant}(151,1,1523)\right\}\\ \left\{\mathrm{Applicant}(11,9,13)\right\}\\ \ldots \ldots \\ \left\{\mathrm{Applicant}(i,j,m)\right\} \end{array}\right.$$

Each piece of the data in the formula is associated with the applicant’s hash. Data are in a three-dimensional representation, where *i* is the rice batch, *j* is the link, and *m* is the specific number of rows in the database. We separately specified these three variables, where *j* corresponds to a total of 13 links in the rice supply chain, and the value of *m* corresponds to the specific position of the required cross-chain data in the nine parallel chains.

The K-means algorithm is an unsupervised learning algorithm that automatically clusters sample data based on the measurement standard of the correlation between data samples. Its objective function is shown in Equation (2):

(2) $$\arg\min\sum_{i=1}^{k}\sum_{t=1}^{n_{i}}{\left\|p_{t}-u_{i}\right\|}^{2}$$

In the formula, *k* is the number of clusters; *n_i_* is the number of sample points in cluster *i*; *p_t_* is the t-th data sample; and *u_i_* is the centroid of cluster *i.* We clustered the sample data based on the Euclidean distance between the sample data and the centroid, as shown in Equation (3):

(3) $$d(t,i)=\sqrt{\sum {\left(p_{t}-u_{i}\right)}^{2}}$$

We designed a secondary clustering method to preprocess the concurrency of cross-chain data. The K-means algorithm design was as follows:

(1) Initial clustering. First, initialize the *k* value according to the preset nine parallel blockchain data storage ranges. The *k* value is the number of *m* across the parallel blockchain in the data packet, which is determined by the maximum and minimum values of *m*. In addition, we determined the corresponding initial test centroid according to the value of *m*, as shown in Equation (4):
  (4) $$c=\frac{m_{1}+m_{2}+m_{3}+m_{4}+\ldots \ldots +m_{n}}{n}$$

The *c* value is the mean value of each part after sorting the values from small to large and dividing them into *k* parts, and clustering the cross-chain data on the value of *m*. The purpose was to form the distribution form of I as shown in Figure 5 for the cross-chain data packet (*Data*) in preparation for the secondary clustering. Different colors in I represent different data requests.

(2) Secondary clustering. According to the similarity of the values of *i* and *j*, two clusters were performed based on the first clustering to form the distribution state of II shown in Figure 5. The different colors in II represent the data classified into different categories after the secondary clustering.

The method of secondary clustering was used to make the data in *Data* clustered when the storage locations of the same or similar batches and links were similar, thereby reducing the number of search calculations.

A Bloom filter is a binary data structure used to determine whether an element is in a set. After the Data were clustered by the K-means algorithm, *k* data blocks were formed. At this time, it was only necessary to verify a random piece of data in each data block to determine the names of the parallel blockchains of all elements in the data block. Specifically, a Bloom filter was designed for each parallel blockchain, and the hash of the data contained in each parallel blockchain was mapped to the Bloom filter in advance. When verification was required, only nine parallel-chain Bloom filters were required to judge the data to be determined, and then specific classifications could be carried out.

When a large amount of data needs to be cross-chained, a time level “*T*” was designed to control the data of each cross-chain. We used the organic combination of the K-means algorithm and Bloom filter to quickly determine the parallel chain where the data were located within *T* time, thereby improving the efficiency of the data cross-chain, and the problem of high concurrency of cross-chain data was resolved.

#### 4.2.3. SPBFT Consensus Mechanism

##### SPBFT Consensus Algorithm

**Figure 6:**
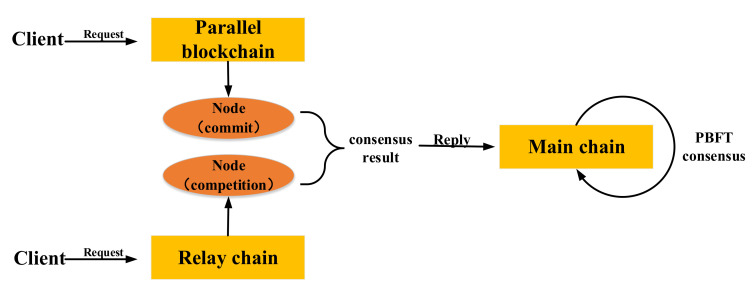
Schematic diagram of SPBFT consensus algorithm.

The consensus mechanism is an important component of blockchain technology. The cross-chain supervision model of the rice supply chain involved the main chain, relay chain, and multiple parallel chains. Reaching a consensus among the main chain, relay chain, and parallel blockchain was the basis for the implementation of the cross-chain supervision of each node in the rice supply chain. PBFT is an election consensus algorithm for a single-chain structure. It is not suitable for multichain consensus. We designed the SPBFT consensus algorithm based on the voting consensus algorithm PBFT. Figure 6 shows the SPBFT consensus algorithm.

We adopted the consensus method of “Chain link Chain” to achieve a consensus among the main chain, parallel blockchain, and relay chain. For the main chain, each supervisory node, enterprise node, and consumer node processed messages through the node status (consistency) of each stage to achieve a complete message-processing state, thereby achieving consensus. For parallel blockchains and relay chains, the SPBFT consensus algorithm was used to realize the migration of the information state from the parallel blockchain to the main chain, and the consistency of the states of the parallel blockchain nodes and the main chain nodes was realized. Finally, “Chain link Chain” → “Node link Node” was realized.

##### SPBFT Consensus Process

**Figure 7:**
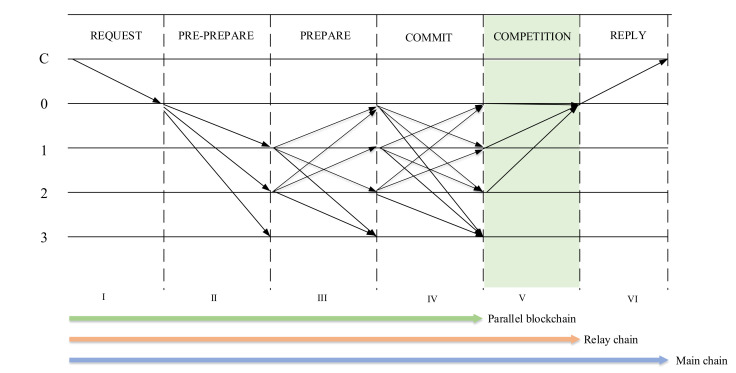
Schematic diagram of SPBFT consensus sequence diagram.

The SPBFT consensus process of the main chain, parallel blockchain, and relay chain selected different consensus steps according to different types of blockchains. Figure 7 shows the SPBFT consensus sequence diagram. The SPBFT had six steps. Compared with the PBFT consensus mechanism, a competition step was added to adapt to multichain consensus. For steps I–VI, the specific descriptions are as follows.

**Request**: Client *C* sends a request to the master node.

**Preprepare**: The master node assigned a unique number *n* to the request, and formed a preprepare message with the request. After signing, the preprepare message was delivered to all member nodes. The unique number *n* consisted of the type, the parallel blockchain number, and the request number. The parallel blockchain number here was only assigned when the parallel blockchain consensus was reached, and the rest were preset to 0, which aimed to solve the multichain consensus concurrency problem.

**Prepare**: When the member node received the preprepare message, it relied on the signature to judge the correctness of the message, and it judged whether to accept it or not. After confirming that it was correct, the signature of this section and *n* were combined to form a prepared message, and the message was broadcasted to all other member nodes.

**Commit**: After receiving the prepare message, all nodes verified the correctness by signing. If the number of prepared messages received by each node exceeded two-thirds of the total number of nodes, it broadcasted a commit message to all nodes, indicating that the node could perform the requested service.

**Competition**: The competition stage was dedicated to the consensus of the relay chain. Our initial definition was that all nodes in the relay chain were trusted nodes. After all nodes received the commit message, every two nodes played a free game. All nodes broadcasted game information, game rounds, and signatures to other nodes, where the signatures were the signature verifications performed by the node that failed the game. After receiving the message, other nodes confirmed the winning node by verifying the signature and the number of signatures. The winning node performed the second round of the game and sent game messages until the only winning node was selected after *N* rounds. The winning node sent the game information and signatures of *N* other nodes to all nodes to form a game message competition.

**Reply**: All nodes, if they received a commit message, verified the correctness of the message. If the number of commit messages exceeded one-third of all nodes, the business of the request was completed, and a reply message was constructed to reply to the client. The client judged whether the system had completed the request based on whether it had received the correct reply from more than one-third of the nodes. For the relay chain consensus, all nodes received the competition message and verified the correctness of the message. The winning node completed the business requested by the request. Then, it constructed a reply message, and it directly replied to the client. The client judged whether the system had completed the request based on whether it had received the correct reply from more than one-third of the nodes.

For the main chain consensus, we assumed that the total number of main chain nodes was *n*, the number of error nodes was *f*, and the number of malicious nodes was *f*. To ensure the security of the consensus, the number of secure nodes needed to be controlled above *f* + *1* nodes. According to Equation (5), the number of nonsecure nodes that the main chain could tolerate was (*n* − 1)/3. The process of the main chain consensus was the traditional PBFT consensus step. The nodes only involved the nodes of the main chain, and did not involve the relay chain and parallel blockchains.

(5) f+f+f+1=n→f=(n−1)/3

For the parallel blockchain consensus, the consensus reached involved each node of the parallel blockchain and each node of the main chain. The specific consensus process was: the client initiated a request message on the main chain, and the main chain broadcasted the message to each node of the parallel blockchain. Each node of the parallel blockchain completed steps I–IV independently, and cross-chains to each node of the main chain after the reply message were constructed in step VI. The reply message was used as the request for the second round of consensus on the main chain, and each node of the main chain would directly reply to the client after reaching a reply-1 message.

Regarding the consensus of the relay chain, the achievement of the consensus involved each node of the relay chain and each node of the main chain. Specifically, the client initiated a request message on the main chain, and the main chain broadcasted the message to each node of the relay chain. Each node of the relay chain completed step IV independently, and after constructing the reply message in step VI, it broadcasted to each node of the main chain. The reply message was used as the request for the second round of consensus on the main chain, and each node of the main chain reached a reply-2 to the client after the message.

## 5. Results and Analysis

### 5.1. Operation Process Analysis

**Figure 8:**
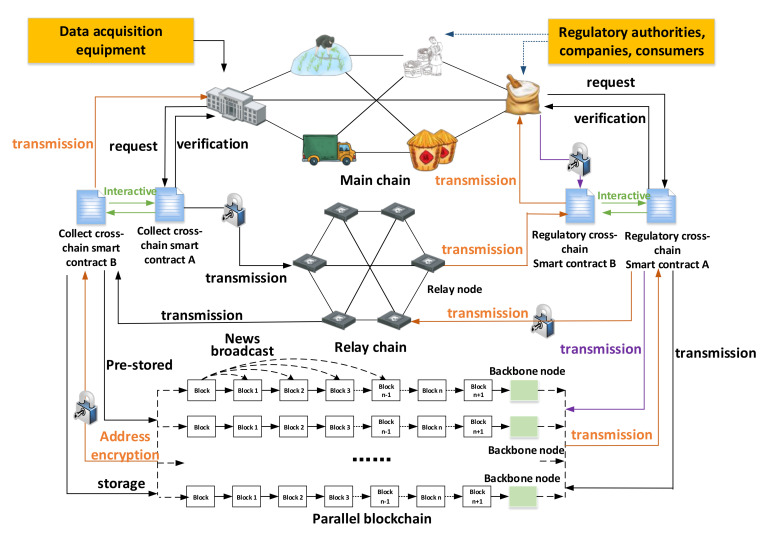
Schematic diagram of mode flow.

The cross-chain model of rice supply chain supervision based on the blockchain and smart contracts fundamentally solved the serious problem of supply chain centralization, and achieved complete decentralization of data. Figure 8 shows the mode flow chart. We analyzed the operation process, and the results showed that the multichain model could effectively realize the supervision of the entire life cycle of the rice supply chain by the supervisory authority.

The cross-chain model of the rice supply chain was divided into cross-chain collection of data and cross-chain supervision of data, and they respectively corresponded to trusted cross-chain storage of data and trusted cross-chain supervision. For a cross-chain collection of data, the participating enterprise nodes of the main chain used data-collection equipment to collect data. The identity of the request initiator was verified by CCSC-A, and hash locking and asymmetric encryption were used to encrypt the data. The fragments were stored in the relay chain. Each node of the relay chain realized the distribution of reorganization rights through the game method. The data summary by CCSC-B was used to prestore the data, and the corresponding storage address was obtained. The address was encrypted by the same hash lock, and mutual decryption of encrypted data was realized through mutual calls between CCSC-A and CCSC-B. Finally, the safe storage of data was realized within the specified time. For the cross-chain supervised data, the main chain supervising node initiated a data call view request, and used SCSC-A to verify its identity. After that, the requested data were preprocessed, and the request was sent to the parallel blockchain. Through SPBFT, the parallel blockchain transmitted the data to SCSC-A, which was hash locked and asymmetrically encrypted by SCSC-A, and then it was fragmented into the relay chain. The winning node of the relay chain game sorted the data and sent it to SCSC-B, which then used the same hash lock to encrypt the identity certification document of the regulatory authority. Then, SCSC-B realized the decryption of the data through mutual calls with SCSC-A. Finally, SCSC-B sent the data to the supervisory department, and SCSC-A stored the invocation information of the supervisory department on the parallel blockchain to realize the on-chain storage of the data interaction records.

This mode could realize the safe storage and call of data between the main chain and the parallel blockchain. It was a completely feasible and credible mode. The model could realize the trusted cross-chain transmission of data among the main chain’s various regulatory department nodes, enterprise nodes, and consumer nodes. Given the complex links, the long life cycle, and the diversity of participants in the rice supply chain, this multichain model could achieve safe, effective, and completely credible supervision of rice quality.

### 5.2. Security Analysis

In the process of data cross-chain transmission, it is easy to encounter malicious attacks, and it is easy to cause cross-chain data to be attacked by Sybil attacks, data tampering, data leakage, data theft, etc. (Table 4).

The cross-chain supervision model of the rice supply chain based on blockchain and smart contracts can effectively resist the attacks mentioned in the table. Regarding the challenge of the consensus mechanism, the SPBFT consensus mechanism was used to achieve a secure consensus on the main chain, parallel blockchain, and relay chain. The parallel blockchain is a storage chain; its purpose is only to share the storage pressure on the main chain. The relay chain itself is a trusted chain, and a notary exists in the form of a blockchain. Therefore, the SPBFT consensus mechanism adopts the “chain link”. It can tolerate less than one-third of invalid or malicious nodes. For a Sybil attack, during the cross-chain process, a cross-chain smart contract is customized. The contract can verify the identity of the main chain node and upload the node’s identity certificate to the parallel blockchain storage, which can effectively prevent the cross-chain process from being attacked. To counter the risk of data leakage, tampering, and loss, a four-fold guarantee mechanism was designed to ensure data security. The first guarantee mechanism was the hash lock mechanism, which encrypted the data by generating a unique hash value through a number generated randomly. Through the time lock and the hash lock, the “counterparty risk” in data transmission was prevented. The second guarantee mechanism was an asymmetric encryption mechanism, which encrypted the data locked by the hash and the hash value through the public key of the applicant, preventing other nodes from using the hash value to “steal” the random number to decrypt the data. The third guarantee was the relay chain mechanism. After the data were encrypted, slices were stored in the relay chain. Each node contained only part of the encrypted data, and the time *T* was set. After the transmission was completed, the data were automatically encrypted and locked. In addition, each node of the relay chain obtained the right to reorganize through the game, which increased the predictable complexity of the position of the reorganized node, thereby ensuring data security. The fourth guarantee was the smart contract mechanism. Through the automatic inspection and nontamperability of the smart contract, the smart contract was customized to verify the identity of the personnel and assist in the credible cross-chain transmission of data.

We conducted experimental simulations on the multichain supervision model of the rice supply chain and adopted a cloud server, which was configured with a four-core CPU, 8 GB of RAM, and a 50 GB high-performance cloud hard drive. The cloud server had 32 nodes, of which 8 were used as main chain nodes, corresponding to the representative enterprises in the six main links of the rice supply chain and two supervisory departments. Among them, six were arranged as relay chain nodes, and the relay chain game adopted CFR (the Virtual Regret Minimization Algorithm). The remaining 18 nodes built three parallel blockchains, and each parallel blockchain deployed six nodes, corresponding to stored corporate information, supervisor information, and cost information. Different malicious nodes and wrong nodes were set up to test the correctness of the cross-chain transmission in the multichain supervision model of the rice supply chain. The test set was divided into three groups, and the number of error nodes in each group was 0, 1, and 3, respectively. Each test set performed 500 cross-chains for data collection and cross-chains for supervision data. Since the parallel blockchain and relay chain nodes were trustworthy, the faulty nodes were deployed to the main chain node for testing. The SPBFT consensus mechanism could effectively tolerate failures below 30% (only for the main chain nodes), and the cross-chain success rate reached 100%, which could fully guarantee the security of data cross-chain transmission.

### 5.3. Efficiency Analysis

The performance of the multichain architecture was tested and analyzed, mainly for cross-chain concurrency issues. For the concurrency problem, this study adopted secondary clustering, and equipped each parallel blockchain with a Bloom filter to solve the cross-chain concurrency.

**Figure 9:**
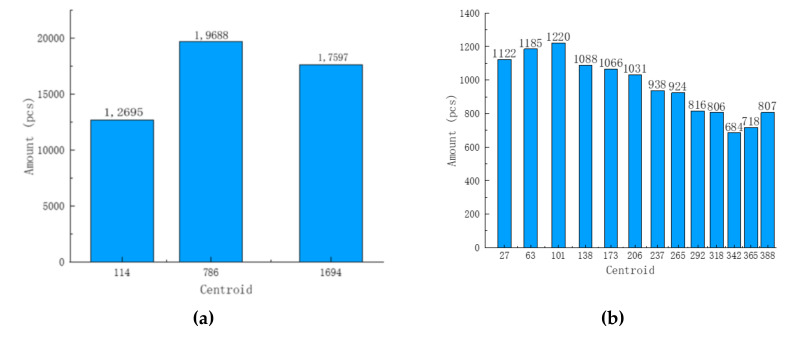
Concurrent simulation. (**a**) First clustering result graph; (**b**) Second clustering results graph.

This study used experimental simulation to transmit 50,000 sets of data at the same time to test the concurrent processing capabilities of the rice supply chain. First of all, the concurrency mechanism performed the first clustering. Figure 9a shows the first clustering situation. The abscissa shows the cluster center of the data, and the ordinate is the number of data. After the clustering was successful, each cluster randomly selected a piece of data to match with the Bloom filter in the parallel blockchain. Screening was performed to determine the parallel blockchain represented by each cluster. The first clustering took 0.60823 s and was iterated six times in total. The results showed that the data could match the corresponding parallel blockchain position after a hash conversion. Secondary clustering of the 12,695 sets of the first chain’s data was performed to facilitate the data search. Figure 9b shows the second clustering situation. The abscissa shows the cluster center of the data, and the ordinate is the number of data. The second clustering took 1.32542 s in total and was iterated 22 times. The results showed that the test data set stored in the first chain were concentrated in the 63rd and 101st batches.

Through a simulation analysis, the mechanism could effectively prevent the occurrence of concurrency. The data filtered by the K-means algorithm and Bloom filter clustering could increase the cross-chain speed and quickly determine the specific location of its storage.

### 5.4. Scalable Analysis

The research on the cross-chain model of rice supply chain supervision based on the blockchain and smart contracts showed that the model has good scalability. It is not only suitable for the supervision of the rice supply chain, but also has universality in the fields of the supply chain, medical care, and finance. Specifically, the cross-chain mechanism based on “hash lock + relay chain + cross-chain smart contract” adds data to cross-chain transmission mechanisms such as the notary mechanism, relay/side chain mechanism, and hash lock mechanism, and the complexity is cracked. It also adds a cross-chain attacked emergency locking mechanism. It is safe and realizes the safe storage and transmission of data, which expands the multichain from the initial asset exchange to other industries. The concurrency mechanism based on the K-means algorithm and Bloom filter can play a good role in improving big data and multirequest situations, and it can effectively deal with the occurrence of concurrency problems. The SPBFT consensus mechanism we designed can achieve consensus among multiple chains, and in a true sense realize the interconnection of multiple chains. This makes SPBFT not only applicable to the rice supply chain, but the consensus can also be extended to other supply chain applications, to better ensure product quality and safety.

### 5.5. Discussion

Since entering the era of blockchain 2.0, researchers have begun to study the application of cross-chain technology in all walks of life. They have studied the use of multichain systems to replace the existing single-chain systems to improve the performance and efficiency of the blockchain. In [10], Bhaskara et al. proposed a novel blockchain-based architecture, Fortified-Chain. They introduced a hybrid computing paradigm of blockchain-based distributed data-storage systems. Their research overcomes the shortcomings of blockchain-based cloud-centric healthcare systems, such as high latency, high storage costs, and a single point of failure. In [13], Peng et al. designed a two-tier blockchain structure for vaccine production regulation that was used to address the shortcomings of traditional centralized management. In [14], Laavanya et al. proposed a blockchain-integrated, privacy-assured IOMT framework for stress management while considering sleeping habits, in which every user was assigned their own private permissioned blockchain to ensure data storage and privacy. We compared and analyzed the information supervision models of the rice supply chain based on parallel blockchains and smart contracts designed in this study, as shown in Table 5.

In terms of security, the authors of [10] designed a three-layer fortified-chain architecture, as well as a patient-anonymization mechanism. The authors used a selective ring-based access-control mechanism, which had a good performance in terms of fault tolerance, attack diversity, security recoverability, attack cost, and efficiency, but its application scenario was limited to the healthcare domain, its scalability was insufficient, and its latency was high. Ref. [13] designed a two-layer blockchain structure, as well as a multinode cooperative consensus mechanism. It had a better performance in terms of fault tolerance, attack diversity, security recovery, attack cost, and efficiency, but its application scenario was limited to vaccine production, its scalability was insufficient, and there were no major improvements in throughput or latency. The authors of [14] designed a multichain storage architecture and RSA encryption mechanism with a POW consensus mechanism, which had a better performance in terms of fault tolerance and efficiency. However, it performed poorly in terms of attack diversity, security recovery, and attack cost; was insufficiently scalable; and had high latency. In this paper, we designed a four-fold guarantee mechanism including hash locking, asymmetric encryption, a relay chain, and a smart contract to resist various attacks, and we designed the SPBFT consensus mechanism, which had a better performance in terms of fault tolerance, attack diversity, security recovery, and attack cost. The model we designed used concurrent mechanisms based on K-means clustering and a Bloom filter, which were more efficient in combination and more scalable. However, the resource consumption of the model designed in this paper was still high compared to the traditional single-chain architecture.

## 6. Conclusions and Future Work

To strengthen the capabilities of rice supply chain supervision under an epidemic, a rice supply chain supervision cross-chain model based on blockchain theory and smart contract technology was designed. Firstly, this study constructed an overall analysis of the rice supply chain, and we abstracted the main links of the entire supply chain. Then, the supply chain data were divided into parallel chains. Secondly, a cross-chain model of rice supply chain supervision was designed. The model included a cross-chain framework for rice supply chain supervision, a cross-chain mechanism based on “hash lock + smart contract + relay chain”, a concurrency mechanism based on K-means clustering and a Bloom filter, and an SPBFT consensus mechanism. Finally, the operation process, safety, efficiency, and scalability of the model were analyzed in this study. The results showed that this model research was an innovative practice in rice regulation by applying the multichain model to rice supervision. In terms of the entire rice supply chain industry, this model strengthened the exchange of information between different links of rice, and realized point-to-point real-time data exchange between different types of enterprises in the rice supply chain. In terms of credible supervision of the rice supply chain, it improved the cohesion of supervisors with the data of the entire rice supply chain. The model strengthened the coupling between rice supply chain data, basic information, harmful substance information, and personnel identity information, and realized the refined management of rice supply chain data and personnel. In terms of the model technology of the entire rice supply chain, the convenience and security of cross-chain data interaction between each link of the rice supply chain was improved, and the storage cost of supply chain data and the high latency of interaction were reduced. We realized the credible supervision of rice data with better distributed ledger technology, ensuring the quality and safety of rice.

A comparison of our proposed model with the traditional centralized management model and other popular blockchain-based methods for managing agricultural and food information is shown in Table 6. In the comparison, it can be seen that our proposed system had a high level of security and a relatively low cost due to the use of parallel chains for data storage and the customized design of smart contracts. The model could automatically and efficiently interact with the data, with less reliance on manual and storage devices.

Since the SPBFT consensus mechanism was established based on the PBFT consensus mechanism, its fault node tolerance was one-third. To solve this problem, we will try to improve the SPBFT consensus mechanism to increase its fault tolerance. In addition, we will explore the combination of the cross-chain model with the identification analysis technology in the industrial internet and the Internet of Things. Applying the cross-chain model to the entire category of grain and oil supply chain supervision and other products’ supply chain supervision will be a direction of our future efforts. The cross-chain supervision model based on the rice supply chain was designed in this paper to apply to the unique links and information flow characteristics of the rice supply chain. After qualitative modifications of the supply chain architecture and information flow characteristics for other categories of cereals, the novel architecture, cross-chain mechanism, and concurrency mechanism proposed in this paper can be applied to the supply chain information management of all categories of cereals. This provides an idea for the information management of the whole grain oil category.

This research provided a feasible and practical solution for accelerating the digital transformation of the food industry that can enhance the ability to supervise food crops and ensure food security.

## Figures and Tables

**Table 1:** Literature review classification table.

Category	Main Content	References
A theoretical study on the framework or model of agricultural products and food management based on the blockchain and smart contracts	Exploring the advantages of blockchain applications in agricultural products and food management frameworks or models	[19,20,25,29]
Research on information management of agricultural products and food based on the blockchain and smart contracts	Focus on changing the traditional centralized management model through the decentralized nature of the blockchain, which is used to strengthen the information control ability of agricultural products and food	[1,24,32,35,38]
Research on traceability of agricultural products and food information based on the blockchain and smart contracts	Improve information traceability of agricultural and food products by using the characteristics of the blockchain such as nontamperability and transparency	[21,22,33]
Blockchain-based applications for the integration of agricultural products and food with the Internet of Things, etc.	The integration of technologies such as the Internet of Things and the blockchain is used to improve the information traceability and management of agricultural products and food.	[17,28,30,34]

**Table 2:** Parallel blockchain data division.

Batch:		
Parallel Blockchain		Key Data
I	Hazard information	Mycotoxins, **heavy metals**, **pesticide residues**, pests, fumigants and herbicide residues, abnormal temperature and humidity, mildew, generated fungi, and toxins.
II	Corporate information	Company name, company address, **company contact information**, **business license**, main business, legal representative, legal person contact information, registered capital, and **enterprise nature**.
III	Consumer information	**Identity information**, contact information, home address, **the purpose of the purchase**, **time of purchase**, **place of purchase**, goods purchased, and **product shelf life**.
IV	Regulatory information	Institution name, department, **supervision link**, link standard description, **rules and regulations**, prevention and control strategies, **supervision data**, supervision progress, information of responsible personnel, **problem product records**, qualified product records, supervision time, and supervision methods.
V	Transaction record	**Purchase price**, purchase source, purchase time, purchase amount of fertilizers, seeds, pesticides, films, etc.; **use of planting equipment**, purchaser information, seller information, real-time purchase price, purchase time, etc., **drying equipment purchase information record**, drying staff salary record, plant expense records, etc., **impurity removal equipment**, drug purchase information records, site expense records, etc., **storage time**, expense information records, **rice batches**, ridge equipment purchase information records, equipment maintenance costs, parts purchase records, rice milling equipment purchases information record, **color sorting equipment purchase information record**, polishing equipment purchase information record, impurity removal equipment purchase information record, worker salary record, **storage time**, expense record; management record, transportation distance record, driver salary record, distance cost record, sales batch second, sales records, and venue rental records.
VI	Cost information	**Seed price**, fertilizer price, labor cost, total cost, sales price, labor cost, **drying** (equipment, etc.) **cost**, **cleaning** (medicine, equipment, etc.) cost, **storage** (warehouse, tools, etc.) cost, storage price, **ridged valley** (equipment, etc.) cost, **rice milling** (equipment, etc.) cost, **color sorting** (equipment, etc.) cost, **polishing** (equipment) cost, packaging cost, primary product price, warehousing cost, outgoing price, transportation cost, driver’s salary, high-speed fee, purchase price, sales price, venue cost, sales staff salary, and publicity expenses.
VII	Data interaction record	**Supervision**, query data records, traceability records, and access records.
VIII	Health record	**Site hygiene conditions**, daily dressing records, daily disinfection records, and cleaning records.
IX	Information	**Seed source**, production site, planting/harvesting time, rice yield rate, fertilizer/pesticide use information, purchase batch, **purchase inspection report**, **drying record report**, pharmaceutical use record, impurity content, impurity removal rate, inventory number, product batch, **product source**, quality inspection report, product category, product quantity, ridged grain method, equipment inspection record, **ridged grain time**, roughness removal/husking rate, **rice milling method**, equipment inspection record, entire rice/broken rice rate, color selection accuracy, carry-out ratio, polishing method, polishing rate, packaging material source, **packaging material qualification certificate**, product quality information, temperature and humidity record report, storage time, storage time, transportation vehicle information, vehicle disinfection report, departure place, route, **arrival time**, driver information, product name, product integrity rate, purchase time, sales time, sales address, and product quantity.

**Table 3:** Permission data table.

Batch:										
Node		Function Permissions								
		I	II	III	IV	V	VI	VII	VIII	IX
Regulatory Authority	National Grain Administration	√	√	√	√	√	√	√	√	√
	Ministry of Finance		√	√	√	√	√	√	√	√
	Ministry of Health	√	√	√	√	√			√	√
	State Administration for Industry and Commerce		√	√	√	√			√	√
	General Administration of Quality Supervision, Inspection and Quarantine	√	√	√	√	√		√	√	√
	Ministry of Agriculture		√	√	√	√		√	√	√

**Table 4:** Attack descriptions.

Attack Type	Description
Consensus mechanism challenge	Whether the consensus algorithm between the parallel blockchain and the main chain can achieve real security.
Witch attack	A malicious node illegally presents multiple identities to the outside world and conducts malicious behaviors after mastering multiple nodes.
Data leakage risk	When data is transmitted between the parallel blockchain and the main chain, malicious nodes attack, resulting in the leakage of data information.
Data tampering risk	In the cross-chain process, malicious nodes attack and tamper with the data during data transmission, resulting in untrustworthy data.
Data loss risk	In the cross-chain process, data is “dropped out”, resulting in data loss.

**Table 5:** Comparative analysis table.

Performance	Index	Ref. [10]	Ref. [13]	Ref. [14]	Our Study
Security	Fault Tolerance	Middle	High	High	Middle
	Attack Diversity	High	High	Low	High
	Security Recovery	High	High	Middle	High
	Attack Cost	High	High	Middle	High
Model Efficiency	Throughout Capacity	High	Middle	Middle	High
	Delay	Middle	Middle	High	low
Scalability	Resource Consumption	High	High	High	High
	Application Scalability	Middle	Low	Low	High

**Table 6:** Comparison of different blockchain application models in agricultural products and food information management.

Category	Information Regulation Method	Labor Cost	Equipment Cost	Security Level
Traditional centralized management model	Manual processing	High	High	Low
Blockchain + InterPlanetary File System (IPFS)	Machine processing	Low	High	Middle
Blockchain + local database	Machine processing	Low	High	Middle
Blockchain + cloud database	Machine processing	Low	High	Middle
This study	Machine processing	Low	Low	High

## Data Availability

The authors declare that the data supporting the findings of this study are available from the authors.

## Appendix A

**Algorithm A1:** Collect cross-chain smart contract A(CCSC-A)
**Input:** *H*(*user*); *D*; *Dn*; DATA; *Ds*; *T*; *AD*; CCSC-B; *pk_p_*; *Y*; *F*; **1: func** Certification(H*(user)*) (*D*) // Data acquisition module { **For** *t* **in** range (m) **If** H*(user)* permissions match Matching collection information:{Hazardous Material Information or corporate information or consumer information or regulatory agency information or transaction records or cost information or data interaction records or health records or other information} **If** Data validation after data standardization **Return** Step 2 } **2: func** Hash lock(D)// Data encryption module { **For** *t* **in** range (*m*) **If** *D* standards compliant Get data summaries *Ds* *D*→*N*→*H(N)// D* takes a random number *N* and generates the corresponding hash value *H(N)* *Dn*←Encrypt *_H(N)_*(*D*)// Encrypt *D* using *H*(*N*) to generate ciphertext *Dn* *DATA*←Encrypt*_pkp_*(*Dn*, *H*(N), *Ds*)// Use the user’s public key to integrate and encrypt *Dn*, *H*(*N*), and *Ds* to generate data packets *DATA* **If** *Ds*, *H (N)*, *Dn*, *DATA generate* **Return** true **Return** false } **3:func** Slice(*DATA*)// Data Fragmentation Module{ **For** *t* **in** range (*m*) **If** Step 2 returns true Slice(*DATA*)//*DATA* Fragmentation Send(*DATA*)→Relay chain _(node)_// Data shards are stored in each node of the relay chain **Return** true **Return** false } **4:func** Game(Relay chain _(node)_)// Game module { **For** *t* **in** range (*m*) **If** Step 3 returns true Game(node)// The game of each node of the relay chain obtains the right of reorganization **Return** Relay chain _(node)_←DATA// Data normalization Each node encrypts data shards and returns *H(DATA)* to the contract **If** Judgment complete: Set(*T*)// Set time lock T Verify(*H*(DATA)>51%)// The amount of *H (DATA)* returned within *T* time is greater than 50% **Return** true **Return** exit } **5: func** Get(*AD*)// storage module { **If** Step 4 returns true Transfer(CCSC-B) **Return** CCSC-A→*N*; AD→CCSC-A// *N* and *AD* exchange to obtain Store *AD* to the blockchain **If** Store successfully verified: Verify(Decrypt (*D*)) →*Y*/*F*// Verification of CCSC-B decryption result notification *Y/F* **Return** Storage(*AD*) and true **Return** Exit and false //main chain, exit is interrupt cross-chain transmission in time }

**Algorithm A2:** Collect cross-chain smart contract B(CCSC-B)
**Input:** DATA; CCSC- A; *N*; *sk_p_* **1:func** Get-Crack (DATA, *skp*)// Obtain DATA and use user *sk_p_* to decrypt to obtain *Ds*, etc. { **For** *t* **in** range (*m*) **If** Preliminary decryption of *DATA:* Get user private key *sk_p_* Decrypt DATA **Return** *Dn*, *H*(N), *Ds* and true } **2: func** Pre-stored(Ds)// Pre-storage based on data summary, get *AD* { **For** *t* **in** range (*m*) **If** data pre-storage: Data preprocessing based on data summaries **Return** *AD* **Return** false } **3:func** Get(*N*)// Obtain *N* through data interaction { **For** *t* **in** range (*m*) **If** Get random number *N*: Transfer(CCSC-A) **Return** CCSC-A→*N*; AD→CCSC-A// *N* and *AD* exchange to obtain **If** decrypt *DATA*: Decrypt with *N* to get *D* Get *H(D)* for *D* hash, verify whether *D* has been tampered with } **4: func** Storage(*D*)// Store according to the pre-stored address { **For** *t* in range (*m*) **If** *Data* storage to parallel blockchain: Store *D* to the corresponding parallel blockchain **Return** Send *Y/F* to CCSC-A }

**Algorithm A3:** Supervise cross-chain smart contract A(SCSC-A)
**Input** *H(S)*; *Ds*; *S*; SCSC-B; *V*; *IR*;*DATA* **1: func** Certification(*H(S)*)// Verify Permissions { **For** *t* in range (*m*) **If** $H\left(S\right)\;permissions\;comply\;with$ **Return** Accept cross-chain requests **Return** false } **2: func** Pretreatment(*D*)// Cross-chain request preprocessing { **If** Data request preprocessing: Request preprocessing based on concurrency mechanism based on K-means algorithm and Bloom filter **Return** The location of the parachain where the requested data resides } **3: func** Integration(*Data*)// Form a supervised cross-chain data *Data* { **For** *t* **in** range (*m*) **If** Cross-chain supervision data processing: Extract each parachain data to form *Data* Generate a random number *N*, take the hash *H*(*N*), use *H*(*N*) to encrypt the *Data*, and get *Dn* Get user *pkp*, encrypt *H*(*N*), *Dn*, get *DATA* **Return** true **Return** false } **4: func** Slice(*DATA*)// *DATA* slices are stored in the relay chain { **For** *t* **in** range (*m*) **If** Relay chain data processing: Set the hash lock *T* Data slices are stored to relay chain nodes Each node of the relay chain plays a game, sorts the *DATA*, and transmits it to SCSC-B **If** *T* ends **Return** The *DATA* fragments of each node in the relay chain are automatically encrypted, and *H(D)* is sent to SCSC-A **If** H(D)>=51% **Return** true **Return** false **Return** false } **5: func** Storage(*IR*)// Interactive record storage { **If** *IR* deal with: *IR:* {Initiate the request related person information, request information, data transmission process information} Stored in the blockchain main chain **Return** ture **else** **Return** false }

**Algorithm A4**: Supervise cross-chain smart contract B(SCSC-B)
**Input***Data*; *N*; SCSC-A; *skp*; *T*; *DATA* **1: func** Decrypt(*DATA*)// Decrypt *DATA* with *skp* { **For** *t* **in** range (*m*) **If** *DATA* processing: Get user private key *skp* Decrypt *DATA*, get *Dn*, *H*(*N*) } **2: func** Get(*N*)// Get *N*, pass *S* { **For** *t* **in** range (*m*) **If** *S* processing: Use the same *H*(*N*) for data encryption **If** data exchange: Transfer(SCSC-B) **Return** SCSC-A→*S*; N→SCSC-B// Exchange *N* and *S* to obtain **Return** false } **3: func** Get(*Data*)// Decrypt to get *Data* { **For** *t* **in** range (*m*) **If** *Dn* processing: Decrypt *Dn* to get *Data* Hash *Ds* to verify whether it has been tampered with **Return** ture **Return** false } **4: func** Transport(*Data*) { **For** *t* **in** range (*m*) **If** *Data* verification without tampering **Return** Regulatory Authority and ture **Return** false } **5: func** Self-defense(*T*, SCSC-A)// Anti-attack design { **For** *t* **in** range (*m*) **If** Step 4 Back ture Send (*V*)→SCSC-A **If** Attack handling: Received the attack information of SCSC-A **Return** *Data* is automatically encrypted }
